# Azithromycin Resistance and Decreased Ceftriaxone Susceptibility in Neisseria gonorrhoeae, Hawaii, USA

**DOI:** 10.3201/eid2305.170088

**Published:** 2017-05

**Authors:** John R. Papp, A. Jeanine Abrams, Evelyn Nash, Alan R. Katz, Robert D. Kirkcaldy, Norman P. O’Connor, Pamela S. O’Brien, Derek H. Harauchi, Eloisa V. Maningas, Olusegun O. Soge, Ellen N. Kersh, Alan Komeya, Juval E. Tomas, Glenn M. Wasserman, Gail Y. Kunimoto, David L. Trees, A. Christian Whelen

**Affiliations:** Centers for Disease Control and Prevention, Atlanta, Georgia, USA (J.R. Papp, A.J. Abrams, E. Nash, R.D. Kirkcaldy, E.N. Kersh, D.L. Trees);; University of Hawaii, Honolulu, Hawaii, USA (A.R. Katz, A. Komeya, J.E. Tomas, G.M. Wasserman, A.C. Whelen);; Hawaii Department of Health, Honolulu (A.R. Katz, G.M. Wasserman, N.P. O’Connor, P.S. O’Brien, D.H. Harauchi, E.V. Maningas, G.Y. Kunimoto, A.C. Whelen);; Gonococcal Isolate Surveillance Project Regional Laboratory, University of Washington, Seattle, Washington, USA (O.O. Soge)

**Keywords:** Neisseria gonorrhoeae, gonorrhea, phylogenic cluster, bacteria, bacterial infection, antimicrobial resistance, genomic analysis, azithromycin, in vitro susceptibility, ceftriaxone, sexually transmitted disease, STD, sexual transmitted infections, STI, Hawaii, United States

## Abstract

During 2016, eight *Neisseria gonorrhoeae* isolates from 7 patients in Hawaii were resistant to azithromycin; 5 had decreased in vitro susceptibility to ceftriaxone. Genomic analysis demonstrated a distinct phylogenetic clade when compared with local contemporary strains. Continued evolution and widespread transmission of these strains might challenge the effectiveness of current therapeutic options.

*Neisseria gonorrhoeae* is a sexually transmitted pathogen that has progressively developed resistance to the antimicrobial agents recommended for treatment (*1*). Third-generation cephalosporins are among the last class of antimicrobial agents that are still effective, and ceftriaxone is the foundation of treatment options recommended by the United States (*2*) and other countries. The diminished cache of drugs to treat gonorrhea has led most countries to recommend a combination of ceftriaxone and azithromycin in an attempt to ensure effective therapy and slow the emergence of resistance by decreasing the likelihood that a *N. gonorrhoeae* isolate would survive concomitant exposure to 2 antimicrobial agents with distinct mechanisms of action (*2*). However, sporadic treatment failures have been reported (*2*), and gonorrhea is considered a global health concern by the World Health Organization and the Centers for Disease Control and Prevention (CDC) because of the few remaining treatment options.

Surveillance for antimicrobial susceptibility of *N. gonorrhoeae* was established by the CDC in the United States in 1986 as penicillin and tetracycline resistance was becoming widespread. The CDC Gonococcal Isolate Surveillance Project (GISP; Division of STD Prevention, National Center for HIV/AIDS, Viral Hepatitis, STD, and TB Prevention) collects ≈5,000 isolates per year from men with urethritis seeking care at sexually transmitted disease clinics across the United States and assesses the isolates for antimicrobial susceptibility (*3*). The findings are used by CDC to formulate national treatment recommendations and develop research and disease intervention priorities. Ceftriaxone remains highly effective in treating gonorrhea in the United States; 99.9% of isolates were inhibited by <0.125 µg/mL in 2014 (*4*). However, the percentage of isolates with decreased azithromycin susceptibility (azithromycin MIC >2 µg/mL) rose sharply from 0.6% in 2013 to 2.5% in 2014. Fortunately, none of the 2014 isolates demonstrated clinical resistance or decreased susceptibility to both azithromycin and ceftriaxone.

The Hawaii Department of Health (HDOH) State Laboratories Division maintains nucleic acid amplification, culture, and antimicrobial drug susceptibility testing by Etest for *N. gonorrhoeae*. During 2016, the HDOH and CDC became aware of several *N. gonorrhoeae* isolates with high-level resistance to azithromycin and decreased susceptibility to ceftriaxone in Hawaii as a result of routine laboratory testing and jointly initiated an enhanced laboratory investigation of the isolates.

## The Study

The HDOH confirmed the identification of 61 isolates of *N. gonorrhoeae*, collected during February 2016–May 2016, and antimicrobial drug susceptibility testing was performed on all of them. Isolates were identified as *N. gonorrhoeae* by using the API NH test kit (bioMérieux, Marcy l’Etoile, France), and the MICs for azithromycin, ceftriaxone, and cefixime was assessed by Etest (bioMérieux) on GC II agar supplemented with 1% IsoVitaleX (bioMérieux). Etest carried out at the HDOH State Laboratories Division found that 8 *N. gonorrhoeae* isolates had extremely high MICs (>256 µg/mL) for azithromycin and MICs of 0.125–0.25 µg/mL for ceftriaxone and cefixime (Technical Appendix). The 8 isolates, which were collected from 7 patients and included 2 isolates (urethral and urine) from the same patient (GCWGS_0182 and GCWGS_0322), were sent to CDC for confirmatory testing using agar plate dilution (*5*).

All 61 *N. gonorrhoeae* isolates were sequenced (paired-end; 2 × 250-bp read length) on an Illumina MiSeq sequencer (Illumina Denmark ApS, Copenhagen, Denmark) at the HDOH State Laboratories Division. De novo assembly was conducted at CDC by using SPAdes 2.5.1 (http://www.cab.spbu.ru/software/spades), and the core genome single-nucleotide polymorphism alignment was generated by using Parsnp 1.2 (http://www.cbcb.umd.edu/software/harvest), with the FA19 genome (GenBank accession no. CP012026) as the reference. The maximum-likelihood phylogeny was reconstructed by using RAxML 8.0.0 (http://sco.h-its.org/exelixis/web/software/raxml) with 1,000 bootstrap replicates. Whole-genome sequencing data were also used to determine the multilocus sequence typing (MLST) and *N. gonorrhoeae*–multiantigen sequence typing (NG-MAST) allelic profiles for the targeted isolates.

Results of the phylogenetic analysis indicated that the 8 isolates were closely related and formed a single clade (Figure) with 223 single-nucleotide polymorphism differences. MLST analysis revealed 1 unique profile, sequence type (ST) 1901 (Technical Appendix), which is a highly successful lineage associated with multidrug resistance that probably originated in Japan (*6*). The results of the NG-MAST analysis indicated that all 8 isolates shared 1 novel profile, ST14121. Epidemiologic investigations did not associate sexual network transmission among the 7 patients, although 2 patients reported sex with the same partner. However, the consistent MLST and NG-MAST profiles, in combination with the strongly supported clade, suggest the circulation of a single strain within the population.

**Figure:**
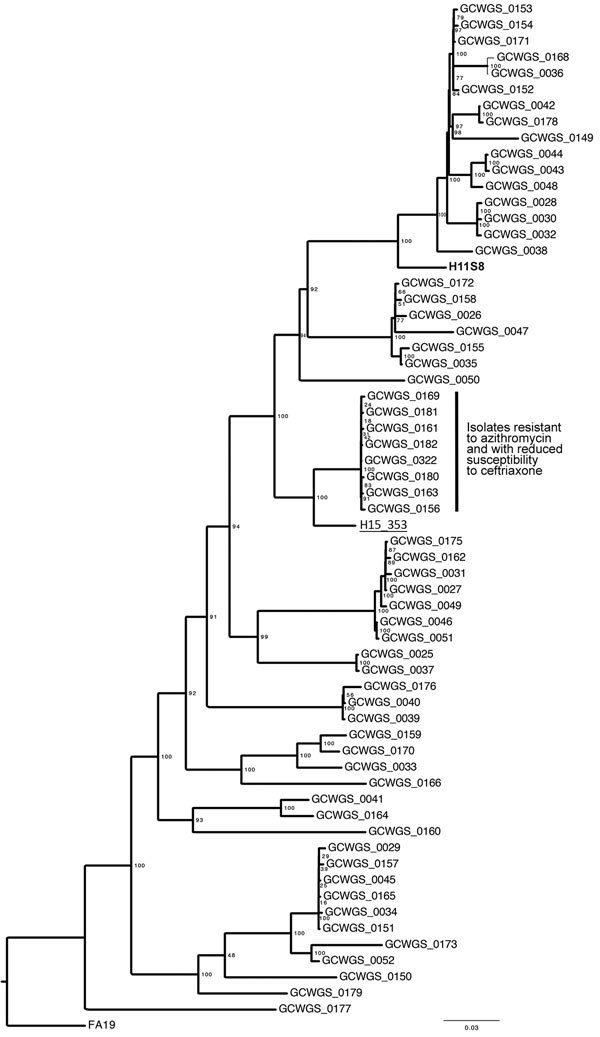
Maximum-likelihood phylogeny of *Neisseria gonorrhoeae* samples (N = 62) collected in Hawaii during February–May 2016, 1 isolate collected in Hawaii in 2011, and 1 isolate collected in the United Kingdom in 2015. The clade denoted with the black vertical bar contains 8 samples that exhibited resistance to azithromycin (MIC >256 µg/mL by Etest) and reduced susceptibility to ceftriaxone (MIC range 0.094–0.125 µg/mL). The 2011 isolate from Hawaii (H11S8, bold) also exhibited resistance to azithromycin. The United Kingdom isolate (underlined) was associated with failed dual antimicrobial therapy of ceftriaxone and azithromycin. The phylogeny is based on the core genome single nucleotide polymorphism alignment of the 62 genomes and the FA19 reference genome. Values on the nodes of the phylogeny (based on 1,000 bootstrap replicates) represent the support for each node and the corresponding clade. Scale bar indicates substitutions per site.

To assess the contribution of known mutations to macrolide and cephalosporin resistance, we examined mutations in *penA*, *ponA*, *mtrR*, and 23S rRNA genes. Regarding azithromycin resistance, a deletion in the *mtrR* promoter associated with low-level resistance (*7*) and 4 mutated 23S rRNA copies with the A2059G mutation that confers high-level resistance (*8*) were identified in all 8 isolates. The *ponA* L421P mutation and mosaic *penA* alleles have been associated with reduced susceptibility to cephalosporins (*7*,*9*). The *ponA* L421P mutation was found in all 8 isolates; however, only the nonmosaic *penA* XVIII allele was detected.

The first *N. gonorrhoeae* isolate (H11S8) with high-level azithromycin resistance (HL-AziR) in the United States was identified in Hawaii in 2011 (*10*). More recently, Public Health England characterized 7 *N. gonorrhoeae* HL-AziR isolates that were collected in northern England during November 2014–March 2015 (*11*). Isolate H11S8 and those from England were more susceptible to ceftriaxone (MIC range 0.004–0.03 µg/mL) than the cluster of *N. gonorrhoeae* HL-AziR isolates identified in Hawaii. Genetic comparisons of the 2011 Hawaii isolate placed it in a distinct clade on the phylogenetic tree (Figure). The NG-MAST of H11S8 was ST649, and those from England were ST9768. Three HL-AziR *N. gonorrhoeae* strains were isolated in 2011 and 2012 in Sweden with slightly higher ceftriaxone MICs (range 0.032–0.064 µg/mL) and were identified as either NG-MAST ST285 or ST8727 (*12*).

All patients infected with the HL-AziR isolates in our study were successfully treated with 250 mg ceftriaxone plus 1 g azithromycin. In contrast, a recent pharyngeal *N. gonorrhoeae* isolate, resistant to azithromycin and ceftriaxone, was recovered from a patient in the United Kingdom following treatment with dual antimicrobial therapy of 500 mg ceftriaxone plus 1 g azithromycin (*13*). Although the isolate was genetically distinct from the 8 isolates in Hawaii, it was more closely related to those 8 isolates than to the other 53 contemporary isolates from Hawaii.

## Conclusions

The combination of ceftriaxone and azithromycin remains the hallmark for the treatment of gonorrhea worldwide on the basis of surveillance data that monitors antimicrobial susceptibility (*2*,*14*,*15*). Slight fluctuations have been observed in ceftriaxone MICs, but rarely have isolates been recovered with a MIC >0.5 μg/mL. However, a growing body of evidence suggests that azithromycin is becoming less effective and should not be used as a monotherapeutic agent for gonorrhea. The observation of increased MICs for ceftriaxone and azithromycin in a cluster of strains from Hawaii might be the harbinger that the effectiveness of current treatment options will be challenged. It is critical that countries expand systematic surveillance for drug-resistant *N. gonorrhoeae* and that laboratories maintain culture capacity to support rapid response activities to confirm suspected treatment failures and mitigate transmission through contact tracing. Expansion of laboratory capacity to conduct genetic analysis in real time would further benefit clinicians and sexually transmitted disease public health programs by identifying novel mechanisms of resistance that could be used to develop nonculture antimicrobial resistance tests and rapidly identify resistant *N. gonorrhoeae* strains in sexual networks.

Technical Appendix Phenotypic antimicrobial susceptibly and genetic strain typing of *Neisseria gonorrhoeae* isolates with high-level resistance to azithromycin and decreased in vitro susceptibility to ceftriaxone, Hawaii.
