# Supplementary material for: Azithromycin Resistance and Decreased Ceftriaxone Susceptibility in Neisseria gonorrhoeae, Hawaii, USA
Source: Emerg Infect Dis. 2017 May;23(5):830–2. doi: 10.3201/eid2305.170088 (PMC5403062; doi:10.3201/eid2305.170088)
Supplement: Technical Appendix — Phenotypic antimicrobial susceptibly and genetic strain typing of Neisseria gonorrhoeae isolates with high-level resistance to azithromycin and decreased in vitro susceptibility to ceftriaxone, Hawaii. [file 17-0088-Techapp-s1.pdf]

Article DOI: http://dx.doi.org/10.3201/eid2305.170088

Azithromycin Resistance and Decreased Ceftriaxone Susceptibility in *Neisseria gonorrhoeae*, Hawaii

Technical Appendix

Technical Appendix Table. Phenotypic antimicrobial susceptibly and genetic strain typing of *Neisseria gonorrhoeae* isolates with high-level resistance to azithromycin and decreased in vitro susceptibility to ceftriaxone, Hawaii

		MIC (MIC, µg/mL) (susceptibility classification)†									
Strain/SRA Accession Number	BLM*	Test type	Azithromycin	Cefixime	Ceftriaxone	Ciprofloxacin	Gentamicin	Penicillin	Tetracycline	NG-MAST	MLST
GCWGS_0156/SRR4048856	(+)	Agar dilution	>16 (R)	0.125 (DS)	0.125 (DS)	16 (R)	8	>64 (R)	2 (R)	ST14121	ST1901
		Etest‡	>256 (R)	0.125 (DS)	0.125 (DS)	ND	ND	ND	ND		
GCWGS_0161/SRR4048862	(+)	Agar dilution	>16 (R)	0.06 (S)	0.06 (S)	8 (R)	4	>64 (R)	2 (R)	ST14121	ST1901
		Etest‡	>256 (R)	0.125 (DS)	0.125 (DS)	ND	ND	ND	ND		
GCWGS_0163/SRR4048864	(+)	Agar dilution	>16 (R)	0.06 (S)	0.06 (S)	16 (R)	4	>64 (R)	2 (R)	ST14121	ST1901
		Etest‡	>256 (R)	0.125 (DS)	0.125 (DS)	ND	ND	ND	ND		
GCWGS_0169/SRR4048869	(+)	Agar dilution	>16 (R)	0.125 (DS)	0.125 (DS)	16 (R)	8	>64 (R)	4 (R)	ST14121	ST1901
		Etest‡	>256 (R)	0.125 (DS)	0.125 (DS)	ND	ND	ND	ND		
GCWGS_0180/SRR4048880	(+)	Agar dilution	>16 (R)	0.125 (DS)	0.125 (DS)	16 (R)	8	>64 (R)	2 (R)	ST14121	ST1901
		Etest‡	>256 (R)	0.125 (DS)	0.125 (DS)	ND	ND	ND	ND		
GCWGS_0181/SRR4048881	(+)	Agar dilution	>16 (R)	0.06 (S)	0.125 (DS)	16 (R)	8	>64 (R)	2 (R)	ST14121	ST1901
		Etest‡	>256 (R)	0.25 (DS)	0.25 (DS)	ND	ND	ND	ND		
GCWGS_0182/SRR4048882	(+)	Agar dilution	>16 (R)	0.06 (S)	0.03 (S)	16 (R)	4	>64 (R)	2 (R)	ST14121	ST1901
		Etest‡	>256 (R)	0.125 (DS)	0.125 (DS)	ND	ND	ND	ND		
GCWGS_0322/SRR5259797	(+)	Agar dilution	>16 (R)	0.125 (DS)	0.125 (DS)	16 (R)	8	>64 (R)	2 (R)	ST14121	ST1901
		Etest‡	>256 (R)	0.125 (DS)	0.125 (DS)	ND	ND	ND	ND		

*Beta-lactamase
†Interpretative criteria for ciprofloxacin (Susceptible ≤0.06µg/mL, Resistant ≥1.0 µg/mL), penicillin (Susceptible ≤0.06 µg/mL, Resistant ≥2.0 µg/mL) and tetracycline (Susceptible ≤0.25 µg/mL, Resistant ≥2.0 µg/mL), cefixime (Susceptible ≤0.25 µg/mL) and ceftriaxone (Susceptible ≤0.25 µg/mL) were in accordance with the Clinical Laboratory Standards Institute. The Gonococcal Isolate Surveillance Project's alert values were used to interpret the MIC values for azithromycin (Susceptible ≤1.0 µg/mL, Resistant ≥2.0 µg/mL). The GISP alert criteria (Decreased susceptible ≥0.05 µg/mL was also used for cefixime and ceftriaxone since CLSI does not report a resistant MIC value. There are no interpretative criteria for gentamicin. R refers to resistant, DS is decreased susceptible, S is susceptible and ND is not determined.
‡Etest MIC values are rounded up to the nearest doubling dilution for comparison to agar dilution results as recommended by the manufacturer.
